# Load Balancing Integrated Least Slack Time-Based Appliance Scheduling for Smart Home Energy Management

**DOI:** 10.3390/s18030685

**Published:** 2018-02-25

**Authors:** Bhagya Nathali Silva, Murad Khan, Kijun Han

**Affiliations:** 1 School of Computer Science and Engineering, Kyungpook National University, Daegu 41566, Korea; nathalis@netopia.knu.ac.kr; 2 Department of Computer Science, Sarhad University of Science and information Technology, Peshawar 25000, Pakistan; murad.csit@suit.edu.pk

**Keywords:** smart home, appliance scheduling, least slack time, load balancing, energy management

## Abstract

The emergence of smart devices and smart appliances has highly favored the realization of the smart home concept. Modern smart home systems handle a wide range of user requirements. Energy management and energy conservation are in the spotlight when deploying sophisticated smart homes. However, the performance of energy management systems is highly influenced by user behaviors and adopted energy management approaches. Appliance scheduling is widely accepted as an effective mechanism to manage domestic energy consumption. Hence, we propose a smart home energy management system that reduces unnecessary energy consumption by integrating an automated switching off system with load balancing and appliance scheduling algorithm. The load balancing scheme acts according to defined constraints such that the cumulative energy consumption of the household is managed below the defined maximum threshold. The scheduling of appliances adheres to the least slack time (LST) algorithm while considering user comfort during scheduling. The performance of the proposed scheme has been evaluated against an existing energy management scheme through computer simulation. The simulation results have revealed a significant improvement gained through the proposed LST-based energy management scheme in terms of cost of energy, along with reduced domestic energy consumption facilitated by an automated switching off mechanism.

## 1. Introduction

The evolution of smart appliances has bolstered the “Internet of Things” (IoT) notion by connecting heterogeneous everyday appliances via the existing networks [1]. Smart devices and appliances in IoT identify and share valuable information without or with minimal human interaction. Expansion of the IoT concept has pioneered novel applications, i.e., smart home, smart healthcare, smart warehouse, smart grid, smart city, etc. [2,3,4,5,6]. The smart home concept was initially proposed to manage rapidly growing smart domestic appliances [7]. However, the general purpose of smart home control systems has deviated to explicit purposes, such as energy management, energy conservation, remote appliance control, etc. Extensive energy demands have drawn the attention of experts in both industry and academia to implement energy efficient smart home applications. Subsequent to drastic energy consumption, the world is looking forward to exploring new realms of energy conservation and generation. Typically, energy consumption of a residential habitat varies with the time of the day, the day of the week, and season of the year. Therefore, Time of Use (ToU) pricing was introduced in a number of scenarios to encourage energy consumers to schedule their loads from expensive peak hours to cost-effective off-peak hours [8,9]. Currently, value-added smart behaviors of domestic appliances are capable of adjusting the execution time to gain monetary benefits of smart metering and varying tariffs [10]. In [11], a web of things based smart control system was proposed for domestic appliances. This work manages energy consumption based on user behavior, despite the load shifting mechanisms and variations in tariff. Novel energy management mechanisms reduce the cost of energy by shaping the usage patterns of appliances to minimize the demand during peak hours. Generation of renewable energies, appliance scheduling, and load balancing play an important role in this regard. Inspired by the smart grid notion, experts have worked on substituting peak electricity demand with small-scale renewable energy generators, e.g., photovoltaic (PV) panels. Thus, it reduces direct electricity usage of domestic appliances.

Load shifting further reduces the electricity demand during peak hours by flattening the load curve. In fact, load balancing is essential for energy optimization of modern smart homes [2]. However, simple load shifting mechanisms can cause adverse effects by creating new peak hours [12]. Hence, a load shifting strategy is beneficial once the peak load is disseminated over several off-peak hours [13,14,15]. In [16], the authors proposed a residential load balancing algorithm to schedule multiple users that share a common source of energy. Minimizing the monetary expense was the main concern of [17], which occupies a game theory to optimize selfish users at the demand-side. Similarly, in [18], loads were shifted to more economical periods considering users’ willingness to shift energy consumption. Nevertheless, frequent interruptions on appliance execution diminish user satisfaction [19]. Thus, load shifting mechanisms with significant monetary optimization and minimum disruption will be worthwhile.

In the recent past, multiple efforts were made to reduce the cost of energy by scheduling domestic appliances. A smart home can schedule its devices and appliances to minimize the cost of energy, with the aid of energy price estimation [20]. In [21], an autonomous appliance scheduler based on time of use (TOU) and real-time pricing (RTP) was proposed for a single home scenario. Similarly, another scheduling mechanism based on dynamic pricing was proposed in [22], which further considers usage patterns of the consumers. Multiple attempts have been made to obtain optimal energy consumption for single-user scenarios. For example, the authors of [16] considered a single-user scenario along with known energy consumption values for all appliances that were included in the experimental setting. Contrastingly, in [23], the authors proposed a scheduling mechanism assuming that operating times of the appliances are known beforehand. In [20], the authors proposed an optimized scheduler for appliances based on neural networks. They occupied a greedy approach to determine the order of energy sources since the proposed architecture consisted of multiple energy sources.

In this paper, we propose a smart home scheduling and load balancing system to minimize energy wastage and improve the performance level. Each appliance operates according to a defined state machine. We assumed that smart domestic appliances act as a random variable with random operational time over a horizon. The load balancing component controls the load of different appliances and the load of the entire house. A scheduling mechanism operates on top of the load balancing constraints while adhering to the least slack time (LST) algorithm.

Section 2 of the article presents a brief overview of similar architectures and methods that are used in appliance scheduling and load balancing. Load shifting models are presented in Section 3. Descriptive information related to the proposed scheme are outlined in Section 4. Section 5 presents the results and discussion of the performance evaluation. Finally, the conclusions are outlined in Section 6.

## 2. Related Works

Recent smart home architectures have focused on improving customer satisfaction, energy usage, and cost reduction since adjusting energy usage patterns can benefit both consumers and grid operators by lowering overall peak demand [24]. However, designing a smart home architecture that satisfies all of the aforementioned goals is a challenging task. Current literature consists of various schemes for energy efficient smart home architectures that consider varying TOU tariff incentives, renewable energy generation, and trading of locally generated energy. In fact, power scheduling and load balancing can have adverse effects on the customer comfort due to frequent interruptions in appliance operation and long waiting times for completion. Hence, scheduling mechanisms should guarantee fewer interruptions and the least possible waiting times.

In [22], an algorithm was proposed to achieve energy efficiency for a reduced monetary expense, while preserving consumer comfort. The scheme was implemented considering uncertainties in operation time, energy consumption of household appliances, and renewable energy generation. The beauty of this uncertainty algorithm is that it reduced 41% of monetary expense on energy compared to a conventional greedy scheduling algorithm. However, the proposed stochastic approach suffered from underestimation and overestimation in best-case and worst-case scenarios, respectively. In [25], the authors proposed a cost minimization approach based on a genetic algorithm. Further, they reduced the peak-to-average ratio (PAR) assuming that cost of energy is high once the predefined power threshold is exceeded. Nevertheless, this experiment considered that appliances operate on a fixed power level throughout their operational cycle. In [26], the authors proposed a scheduling algorithm for appliances that operate over a communication network. The communication-based algorithm reduced the monetary expense on energy considering uniform power supply and wind power generation. The scheme did not consider constraints on starting and ending times for the execution of domestic appliances. In [27], the authors proposed a demand-side scheduling scheme for domestic appliances considering PAR. Initially, the scheme optimized the power usage and operation time of shiftable and throttleable appliances. However, in this study, the uncertain nature of the appliance operation was not taken into account. In [28], the authors aimed to provide uninterrupted power supply to homes while flattening the load profile of the grid. The power distribution was decided upon the energy demand from houses, the remaining energy at the grids, and other energy generating sources. Although the load profile was flattened, this scheme failed to address the load scheduling problem.

As stated before, an algorithm for residential energy scheduling in smart grids was proposed in [16] using game theory. This work presumed the energy consumers were self-interested and selfish. Thus, the authors proposed a distributed demand management strategy. They solved the game model using the best response method on current power load and current tariff. Nevertheless, on the other hand, a frequent load shifting approach can have a negative impact on customer satisfaction. Another approach for smart home scheduling based on RTP was proposed in [29]. The authors proposed a versatile convex programming for autonomous load balancing of domestic appliances. They occupied RTP instead of estimated pricing or day-ahead pricing to alleviate the deviation from the actual cost of energy. Since the approach converted the problem into a convex programming (CP) problem, the optimization problem remained convex. Hence, deriving a solution is more efficient. Nonetheless, the generalizability of this framework, which was originally designed for a single house scenario, has not been evaluated. In [30], the authors occupied an optimal stopping approach to propose a real-time demand response (DR) based on the domestic appliance scheduling algorithm. This work aimed to minimize the cost of energy for a variety of household appliances that have different duty cycles. The scheme operated in two stages. Initially, the appliances were scheduled based on the optimal stopping approach and disregarding power constraints. Secondly, the power constraints were taken into account. However, demand leveling was least considered in this framework.

In [21], the authors proposed a smart home scenario where the home setting acts as a consumer as well as a producer. The residential energy management system consisted of a rooftop PV system that provides connection to the grid line with minimum disruptions to consumer loads. Even though this work covers appliance scheduling and renewable energy generation, the whole framework was designed for a single home scenario. The extendable nature of the framework was not discussed sufficiently. The authors of [31] proposed a scheme for automatic load scheduling that minimizes the cost of energy. This work discussed the scheduling of controllable and uncontrollable appliances and storage handling in centralized and distributed manners. However, the scenario concerned a single household, which hinders the actual implementation of the framework. Moreover, frequent disruptions during controllable appliance scheduling may increase the customer discomfort level. In [24], an appliance-scheduling scheme was proposed, exploiting time-variable pricing of energy, which is supported by two-way communication of a smart grid. The authors considered flexibilities of thermal and non-thermal appliances to minimize cost with the aid of a model predictive control (MPC) approach.

In summary, the above literature reveals some important challenges that should be addressed in smart home energy management systems. Firstly, the appliance scheduling should be considered in single house aspect to community level aspect so that peak demand handling will be efficient and accurate. This will smooth the PAR curve instead of shifting the peak to another time dimension. Secondly, appliance scheduling should consider the load characteristics, whether the load is schedulable or uninterruptible. Thirdly, the appliance scheduling should be completed with minimal discomfort to the consumer. Finally, the appliance scheduling and load shifting should minimize the cost of energy. Therefore, herein we propose a smart home appliance scheduling scheme that operates on an LST algorithm to minimize the cost of energy while serving the users at the earliest possible time frame with the aid of priority mapping and heuristic function value. To further extend the benefits of this scheme, we introduce an automated appliance switching off system that reduces unnecessary energy wastage.

## 3. Load Shift Models

### 3.1. Appliance Loads

**Figure 1:**
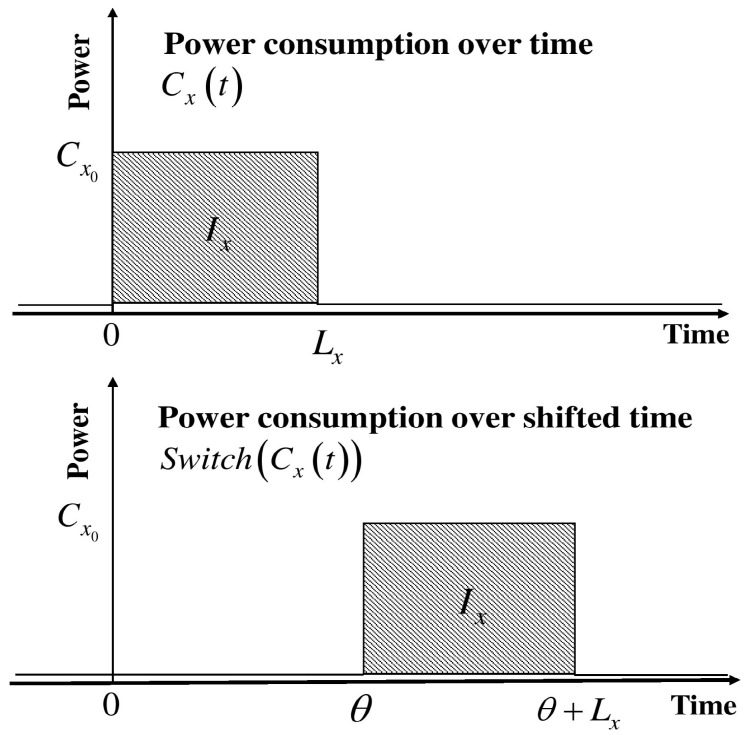
Shifting behavior of the generic load shift model.

Each domestic appliance belongs to one of the three types of load shift models (LSMs): generic load shift model (GLSM), flexible load shift model (FLSM), and periodic load shift model (PLSM). Appliances that initiate operation manually belong to GLSM. In GLSM, the appliances serve a certain flexibility in load shifting. However, the supported flexibility is limited. Dishwashers and washing machines belong to the GLSM category. Figure 1 illustrates a typical operational cycle of GLSM [32]. The power consumption of an appliance during time t is denoted by $C_{x}\left(t\right)$. Power consumption on a particular state is denoted by $C_{x_{0}}$, and O(t,L) stands for an objective function of time period L, which starts from t=0. Switch(f,θ) determines the shifted/switched duration of function f(t) by θ.

(1) $$C_{x}\left(t\right)=C_{x_{0}}\left(t,L_{x}\right),$$

(2) $$Switch\left(C_{x},\theta \right)\left(t\right)=C_{x_{0}}\left(t-\theta ,L_{x}\right),\;\mathrm{where}\;0<\theta <D_{x_{\max}},$$

Energy consumption of an appliance per single execution cycle is determined by $I_{x}=C_{x_{0}}L_{x}$, which is characterized by the appliance energy consumption for the current state $I_{x}$, time duration for the power consumption $L_{x}$, and maximum permitted delay $D_{x_{\max}}$. FLSM adds additional time on scheduling flexibility. Therefore, power load shifting has more controllability over appliances’ power consumption routines. In this load shifting model, a series of delay durations $\bar{\theta }$ are required. Consequently, it models shifting according to the series of the length of delays $\bar{l}$. As in (1), we can model the switching function occupying $\bar{\theta }$ and $\bar{l}$.

(3) $$Switch(C_{x}\bar{\theta },\bar{l})=\sum_{i=1}^{n}C_{x_{0}}O\left(t-\theta_{i},l_{i}\right)$$

where,

(4) $$\theta_{i}+l_{i}<\theta_{i+1},\;\theta_{n}+l_{n}<D_{x_{\max}}\;\mathrm{and}\;\sum_{i=1}^{n}l_{i}=L_{x},\bar{\theta }=\left(\begin{array}{l} \theta_{1}\\ \theta_{2}\\ \cdots \\ \theta_{n} \end{array}\right),\;\bar{l}=\left(\begin{array}{l} l_{1}\\ l_{2}\\ \cdots \\ l_{n} \end{array}\right),$$

**Figure 2:**
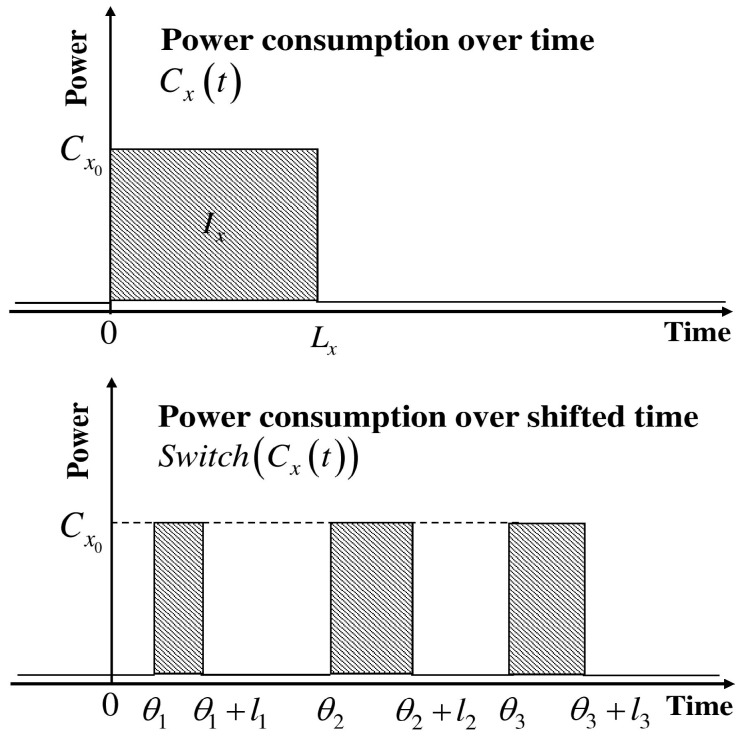
Shifting behavior of the flexible load shift model.

Moreover, FLSM defines an additional parameter $l_{x}{}_{\min}$. In fact, $l_{x}{}_{\min}$ defines the minimum value for the length of the delay period with $l_{i}\geq l_{x}{}_{\min}$. Further, FLSM satisfies $C_{x}=\sum_{i=1}^{n}C_{x_{0}}l_{i}=C_{x_{0}}\sum_{i=1}^{n}l_{i}=C_{x_{0}}L_{x}$. Figure 2 depicts the typical behavior of FLSM [32].

**Figure 3:**
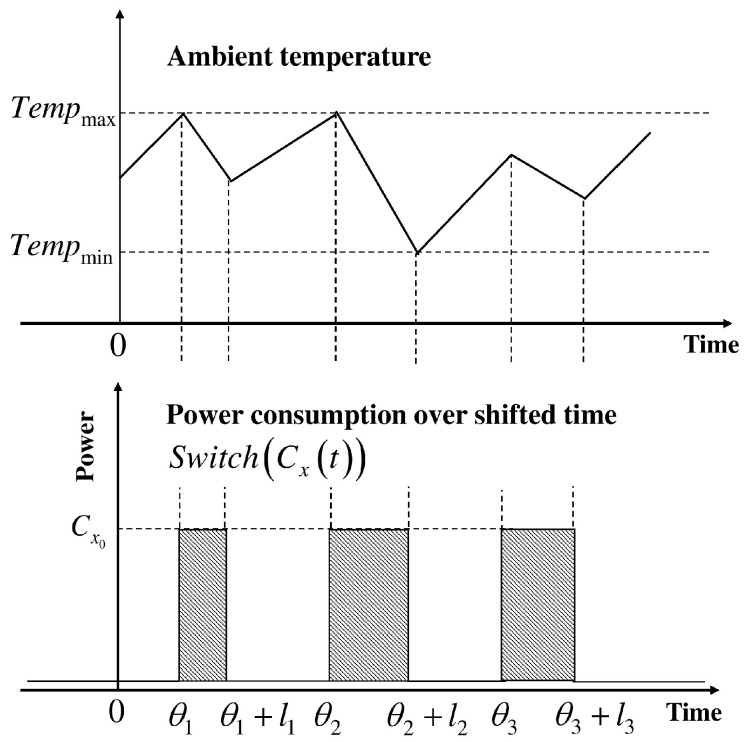
Shifting behavior of the periodic load shift model.

The appliances with periodic operation belong to PLSM. Periodic shifting is allowed when power consumption constraints are observable. For example, coolers operate periodically between maximum temperature and minimum temperature. However, this periodic behavior can be disturbed by human interference, which results in an uncontrollable load. In a typical cooling system, the cooler is turned on when the internal temperature has reached the maximum temperature constraint. Similarly, the cooler is turned off once the ambient temperature is at the minimum constraint. Consequent to heat transfer from the external environment, the ambient temperature fluctuates continuously. Figure 3 illustrates the typical behavior of PLSM with minimum and maximum constraints on ambient temperature. Therefore, these appliances present steady duty cycles in on state $D_{x}{}_{on}$ and off state $D_{x}{}_{off}$. When the power consumption of a particular state is $C_{x_{0}}$ and average power consumption is $C_{x}$, time related average power consumption is as below.

(5) $$C_{x}\left(t\right)=C_{x_{0}}\sum_{i=-\infty }^{\infty }O\left(t-i\left(D_{x}{}_{on}+D_{x}{}_{off}\right),D_{x}{}_{on}\right),$$

(6) $$\bar{C_{x}}=C_{x_{0}}\frac{D_{x}{}_{on}}{D_{x}{}_{on}+D_{x}{}_{off}},$$

PLSM is characterized by on-state power consumption $C_{x_{0}}$, timings for on $t_{x}{}_{on}$ and off $t_{x}{}_{off}$ cycles, and constraints on the minimum duration for on cycle $d_{x}{}_{on}$ and off cycle $d_{x}{}_{off}$. 

The previously stated load shift models are derived from demand-side management (DSM) techniques that aim to reduce peak load and cost. Load shifting is highly recognized as one of the best load management techniques that manipulate peak and off-peak loads [33]. Consequently, experts in both industry and academia have evaluated various load shifting mechanisms including GLSM, FLSM, and PLSM in their works to achieve peak load reduction and monetary benefits [30,32,33,34].

### 3.2. Cumulative Load Management

Delivering a pre-agreed load for a pre-defined period is one of the approaches that are used to manage load shifts. A collection of various domestic appliances facilitates a higher flexibility in load shifting considering time and load parameters. Switching on and switching off loads can provide a significant control over the load in terms of time. Thus, it enhances the power regulation precision of appliances. Nevertheless, the previously stated load shifting approaches do not consider dynamic load properties of appliances. Henceforth, aggregation is introduced to reflect the dynamic load shift properties.

(7) $$Aggr\left(t\right)=\sum_{i=-\infty }^{\infty }{\bar{C}}_{x_{i}}g\left(t-\theta_{i},{\bar{P}}_{i}\right),$$

(8) $$aggr\left(\omega \right)=\sum_{i=-\infty }^{\infty }G\left(\omega ,{\bar{P}}_{i}\right){\bar{C}}_{x_{i}}E^{a\omega \theta_{i}},$$

Assume $g\left(t,\bar{p}\right)$ is the power normalized function, which is applicable to the aforementioned models. Smart energy parameter excluding overall delay is denoted by $\bar{p}$, $G\left(\omega ,\bar{P}\right)$ is Fourier transform of $\bar{p}$, ${\bar{C}}_{x}$ is on-state power model, Aggr(t) is the aggregation, and Fourier transform of aggregation is denoted by aggr(ω). It is clear from (7) that spectral controllability is a linear summation of $G\left(\omega ,{\bar{P}}_{i}\right)$ for the aggregated loads.

## 4. System Architecture

**Figure 4:**
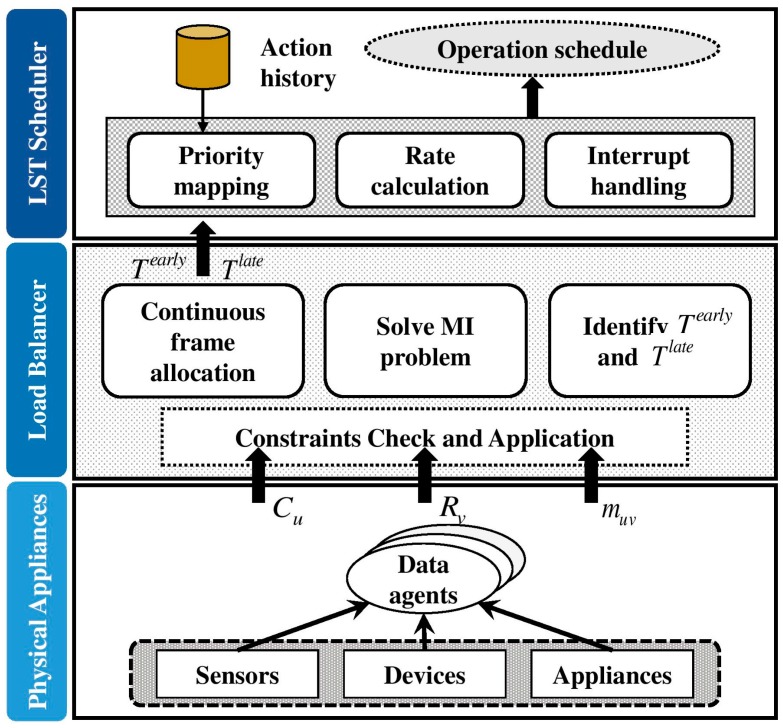
Overview of the proposed load balancing scheme and least slack time (LST)-based scheduling scheme for energy management.

The load balancing part is residing at appliances to control load, incorporating various states and context of home appliances. Similarly, the scheduling mechanism runs on top of the load balancing module incorporating the LST algorithm to efficiently schedule appliance operations in runtime. In order to control energy pricing and consumption states of an appliance, various strategies such as load on demand pricing, time of use, etc., are handled through the smart meter functionality of data agents. The data agents gather various information such as energy pricing and the level of consumption to perform its operations. This information is provided by the smart meter in order to control the functionality and the execution time of an appliance. Each appliance is connected to the Smart Appliance Management System (SAMS) at the physical appliances layer. Data agents are a part of SAMS. The key functionality of SAMS is to reduce energy wastage. Hence, each appliance is attached to a dedicated actuator to execute automated switching off commands from SAMS. Turning on an appliance is a user-initiated random activity. The responsibility of SAMS is to switch off unnecessary appliances when the user is no longer available on the premises. User availability is periodically checked by SAMS to generate control commands for appliances. Worthy to note is that SAMS does not have authority over appliances that operate independently of user presence or user behavior, i.e., dishwashers, EV, and washing machines. Figure 4 illustrates the overview of the proposed smart home energy management system.

Sensors, devices, and appliances constitute the physical appliance layer of the proposed architecture. As stated before, all these sensors, devices, and appliances are monitored and controlled via data agents in SAMS in order to achieve energy efficiency and cost reduction. Data agents determine power consumption $C_{u}$, cost per time unit $R_{v}$, and the requirement for continuous frame allocation $m_{u}{}_{v}$. The load balancer checks the constraints mentioned below in Section 4.2 and performs continuous frame allocation according to the data received from the physical appliance layer. Considering the available task capacity and deadline, the load balancer solves mixed-integer programming problems and subsequently transfers $T^{early}$ and $T^{late}$ information towards the LST scheduling layer. The LST scheduler occupies previous action records to perform priority mapping of the schedule. Once the rate calculation is performed with the received information, the scheduler generates the optimal operation schedule. Moreover, the proposed LST scheduler effectively manages interruptions owing to state transferring of appliances. The key disadvantage of LST in a multiprocessor environment is missing deadlines. The rationale behind this deficiency is LST’s tight coupling (intense dependency) with slack time. However, we introduced an approach to mitigate this drawback with the aid of a priority factor. Priority mapping of the LST scheduler is based on previous action records. Accordingly, the priority factor identifies the jobs to be migrated and the scheduler does the job transferring accordingly. Hence, the job migration process seamlessly manages the release times of each task. Consequently, the LST scheduler can handle multiprocessor requests without missing deadlines of accepted jobs as well as incoming jobs.

### 4.1. Modeling of Smart Home Appliances

**Figure 5:**
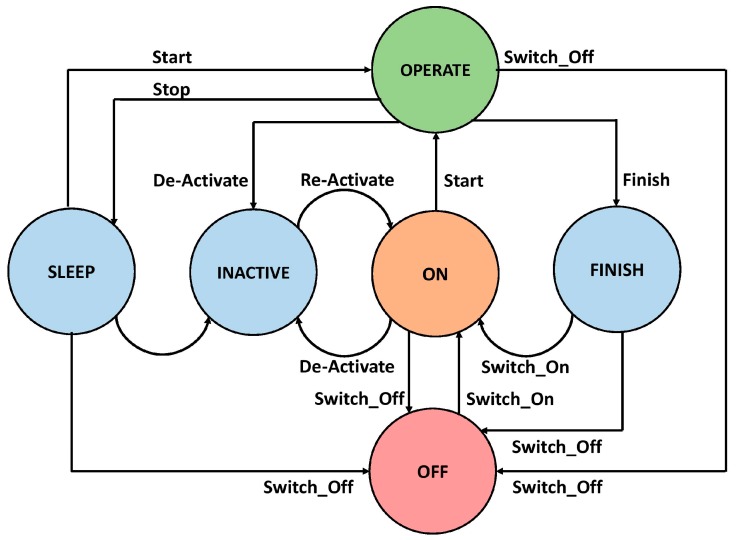
State chart diagram for domestic appliance scheduling.

The smart home appliances act as random variables with random operational time over a horizon. Therefore, we represented these appliances by a finite state machine as shown in Figure 5. The proposed model presented a complete structure of appliance operations including START state and FINISH state. In general, an appliance can have many different states depending on the manufacturer specifications and user context. However, START and STOP are the most common states among all appliance operational mechanisms. Further, the manufacturer also provides the functionality of manually switching off and on an appliance using SWITCH ON and SWITCH OFF state. The manufacturer also provides many constraints to handle the functionality of an appliance. It includes the “Status”, “Preemption”, “Required Energy”, “Power Load”, and so on. However, to be specific, we defined several states that are required to control operations, scheduling, and load balancing of various appliances. The states and descriptions are presented in Table 1.

Current literature reports are rich with real-time scheduling algorithms. However, smart home appliance control and management with time and energy consumption constraints can be solved through proposer selection of the appropriate scheduling algorithm. For example, in a recent work, spring algorithm has been used to handle the home appliance scheduling issues. However, spring has many limitations, for example, it has a complexity of O(nxn!) in many scenarios. Therefore, researchers need to modify the algorithm to fit the home appliance scheduling problem. Further, it cannot guarantee efficient working in many cases, such as handling interruptions, etc. In addition, it has poor performance in real-time scenarios and can lead to several problems in a smart home emergency and surveillance scenarios. Therefore, in this work, we used the LST scheduling algorithm to address the issues present in spring and other scheduling algorithms used for similar purposes. However, the periodic nature of smart home appliances is always a problem and it must be handled with great care and properly adjusted to the different scenarios of a smart environment.

Further, the LST does not perform prior assumption on the event rate. Furthermore, one of the main weaknesses of LST is that it cannot look ahead while working on the current job. That is why LST has poor performance in handling the interruptions. However, we handle such scenarios by modifying the state diagram of an appliance as shown in Figure 5. The interruptions are handled by switching the current job of an appliance from an OPERATIONAL state to either a SLEEP or INACTIVE state depending on the time to perform the rest of the operation and the severity level of the incoming interruption. However, in this work, we did not define the severity or priority level of the interruptions. Further, every interruption is considered as a Boolean variable with value always true. This way the LST can perform 100% in various smart environments.

### 4.2. LST Scheduler

The proposed LST-based scheduler performs three main tasks: priority mapping, rate calculation, and interruption handling. For task priority mapping, the LST scheduler refers to historic operation records. Once the priorities are defined, the scheduler calculates the cost of operation based on the expected energy consumption. Hence, the maximum energy consumption threshold is defined to gain monetary benefit. Upon arrival of a new task request, the scheduler evaluates the expected energy consumption against the maximum energy consumption threshold. The set of task requests are ordered in descending order of heuristic value $H_{T}$. The heuristic function is introduced to ensure computing traceability of the algorithm. A heuristic function is applied on each request k that remains to be scheduled. Since we propose LST scheduling, $H_{T}$ is a function of deadline $d_{i}$ and earliest available time $a_{i}$. Priority is α and γ is a weighted parameter that is constant for a certain environment.

(9) $$H_{T}=\alpha .d_{i}+\gamma .a_{i}$$

If the tasks are non-preemptive and currently at the OPERATIONAL state, the LST scheduler opts to continue its operation. If the tasks are preemptive, task operation feasibility is decided by the maximum energy consumption threshold. If the power consumption of a currently operational task adheres to the maximum power consumption threshold, the LST scheduler continues its operation. Similarly, if a task can complete its work within a maximum power consumption threshold value, LST accepts the incoming task and schedule for operation.

Most of the algorithms in the literature possess uniprocessor capabilities. LST is also a uniprocessor based scheduling algorithm. Nevertheless, we modified the working of LST to work with multiprocessor scenarios such as smart homes in a community, smart city, and smart hospital. The basic LST always has a problem of missing the deadlines due to its dependency on slack time or release time. The multi-job processing functionality is added by migrating jobs based on the priority of an appliance or job as shown in the state diagram. The priority of an appliance is handled by the previous working time of an appliance. Thus, the rate (R) between remaining time and execution time is used to handle the idle state problem. Equation (10) shows the working of the modified LST scheduler.

(10) $$R\left(Job_{i}\right)=\frac{E_{i}^{r}}{TTF_{i}-T}$$

where T denotes the current time of a task i, $E_{i}^{r}$ represents the remaining execution time and $TTF_{i}$ represents the time to finish a job at time T.

Each appliance is assigned a maximum time to complete during a time T. However, if a job does not complete in the maximum allowable time, the job is removed from the operational state and is moved to either a SLEEP or INACTIVE state in order to handle the incoming jobs. Thus, in this way, the modified LST returns optimal results in a smart environment. A modified LST-based scheduling algorithm (Algorithm 1) for domestic appliances is presented below.

**Algorithm 1** Algorithm for appliance scheduling**Variables**: R: collection of requests  k: request where k∈R I(k): theoretical appliance power consumption corresponding to request k pwr: total power consumption of accepted requests  L: maximum power consumption threshold **Goal**: Initialize the request set (R) in descending order of heuristic value   pwr=0
  **for all**
k∈R
**do**   **if** task is non-preemptive and operational **then**
    *accept request*
k
    *remove request*
k from R
    pwr=pwr+I(k)
   **end if**
  **end for**
  **for all**
k∈R
**do**
   **if**
pwr+I(k)≤L and request k is operational **then**    *accept request*
k
    *remove request*
k from R
    pwr=pwr+I(k)
   **end if**
  **end for**
  **for all**
k∈R
**do**
   **if**
pwr+I(k)≤L
**then**    *accept request*
k
    *remove request*
k from R
    pwr=pwr+I(k)
   **end if**
  **end for**

### 4.3. Load Balancing

The load balancer is liable for the distribution of electric load among various tasks in order to schedule the tasks that are denied by the scheduler. Accordingly, it minimizes the cost function associated with the energy price. Each trigger on the load balancer solves a mixed-integer programming problem, such that it minimizes the cost function adhering to the deadline and consumption capacity of the task.

Each task is assigned with an appropriate number of continuous time frames. In load balancing, two main assumptions are made: (1) The power consumption load for each appliance operation is given for its scheduled time period; (2) The demand response module provides necessary information on energy price and power limits.

We presumed a scenario with n appliances and a period of m time frames. Corresponding sets for appliances and time frames are denoted by N={1,…,n} and M={1,…,m}, respectively. Let $x_{uv},u\in N,v\in M$ be a binary variable that represents the status of the appliance, i.e., ON and OFF with value 1 and 0, respectively. Assume $C_{u}$ is the power consumption of appliance x and cost per time unit is denoted by $R_{v}$. Hence, $Cost_{u}{}_{v}=C_{u}R_{v}$ calculates the cost of energy for appliance x to operate over a time frame of v. We introduced an additional variable $m_{u}{}_{v},\forall u\in N,\forall v\in M$ to distinguish the appliances that require contiguous time frames for operation. When $m_{u}{}_{v}$= 1, it ensures the allocation of continuous time frames. Generally, we can define a startup cost $S_{u}{}_{v}$ for each $m_{u}{}_{v}$. This scheme adheres to capacity constraints and balances and schedules the electric load over a time period with the utmost goal of minimizing cost of energy. We attained this goal by solving the following mixed-integer programming problem:

(11) $$\min\;\sum_{u,v}Cost_{u}{}_{v}x_{u}{}_{v}+\sum_{u,v}S_{u}{}_{v}m_{u}{}_{v},$$

(12) $$s.t.\;\sum_{u}C_{u}{}_{v}x_{u}{}_{v}\leq L_{v},\forall v\in M,$$

$$m_{u}{}_{v}\leq x_{u}{}_{j}$$

(13) $$j=j,j+1,\ldots j+\tau_{u}-1,\forall v\in M,\forall u\in N,$$

(14) $$\sum_{v}m_{u}{}_{v}=1,\forall u\in N,$$

(15) $$x_{u}{}_{v}=0,\forall u\in N,\forall v\notin \left(T^{early},T^{late}\right),$$

(16) $$m_{u}{}_{v}\geq 0,\forall u\in N,\forall v\in M,$$

(17) $$x_{u}{}_{v}\in \left\{0,1\right\},\forall u\in N,\forall v\in M,$$

where $L_{v}$ denotes the available capacity for time frame v and $\tau_{u}$ calculates the number of continuous time frames required for appliance u that operates with $Total_{u}$ total energy. Earliest and latest possible start times for appliance u are represented by $T^{early}$ and $T^{late}$, respectively. Furthermore, we considered four sets of constraints in solving this problem as shown below.

(18) $$\tau_{u}=\left\lceil \frac{Total_{u}}{C_{u}}\right\rceil ,$$

- **Constraint 1:** Total power consumption of a particular time slot should be limited to the allotted load capacity.
- **Constraint 2:** Allocate consecutive time frames to each request in all possible scenarios; this provides sufficient operational time and to finish the operational cycle within the deadline.
- **Constraint 3:** Schedule each task once only. Worthy to note that schedule number is subject to changes based on its requirements, characteristics, and priorities.
- **Constraint 4:** Schedule each task in the applicable operation period, such that it assures operation during a specific time period.

The first set of constraints ensures the load capacity of time frame v is always below the stated capacity limit $L_{v}$. The next set of constraints enforces the allocation of $\tau_{u}$ continuous frames for $x_{u}{}_{v}$ when $m_{u}{}_{v}$ value is set to one. Accordingly, the best time frame v to start appliance u between $T^{early}$ and $T^{late}$ to continue operation of $x_{u,v},x_{u,v+1},\ldots x_{u,v+\tau_{u}-1}$ is decided by the optimizer. The third set of constraints guarantees that each task is scheduled only once. The final set of constraints serves conformance to appliance execution within the allocated period by assigning $x_{u}{}_{v}$ to zero in time frames before $T^{early}$ and after $T^{late}$. The beauty of the load balancing scheme is it manages the total power consumption of all manageable appliances. The proposed load balancing mechanism is appropriate for time-dependent appliances with variable power demands, i.e., heating, ventilation, and air conditioning (HVAC) and electric vehicle (EV) charging.

## 5. Results and Discussion

A dramatic increase in the domestic energy demand has motivated experts to implement energy efficient domestic appliances and environments. Even though appliance scheduling is incorporated in many proposed works, the applicability is hindered by the user comfort level. Thus, we have proposed a user-friendly LST-based scheduling mechanism that operates on cumulative load balancing. The performance is evaluated using C# programming language. The smart home environment is designed with multiple smart home appliances and smart home users. The users of the smart home randomly switch on appliances. User activity initiation is a random variable since a prior prediction of user behavior is not feasible. An automated switching off component monitors user behavior and generates control signals accordingly. Simultaneously, load balancing and appliance scheduling algorithm manage power consumption levels of the household to minimize monetary cost of energy. All electronic appliances of the smart home environment were tested for a duration of 1 h. One hour simulation replicated ToU patterns of a single day. Energy consumption of appliances was evaluated as a random variable between 5 to 30 s. In this section, we evaluate the performance of the proposed scheme in terms of energy utilization and cost reduction. Energy utilization results obtained from the automated switching off system and cost reduction attained with the LST-based scheduling are elaborated on in the following sections. The results of the proposed scheme are compared with the results obtained from the scheme proposed in [2]. The scheme in [2] proposed a ZigBee coordinator-based smart energy management system. It manages the operation of household appliances based on previous records. However, the above mechanism does not support load balancing-based scheduling of appliances. Moreover, the scheme of [2] does not consider monetary benefits gained from smart home energy management. Henceforth, we proposed scheme schedules of smart home appliances on the basis of an LST algorithm.

**Figure 6:**
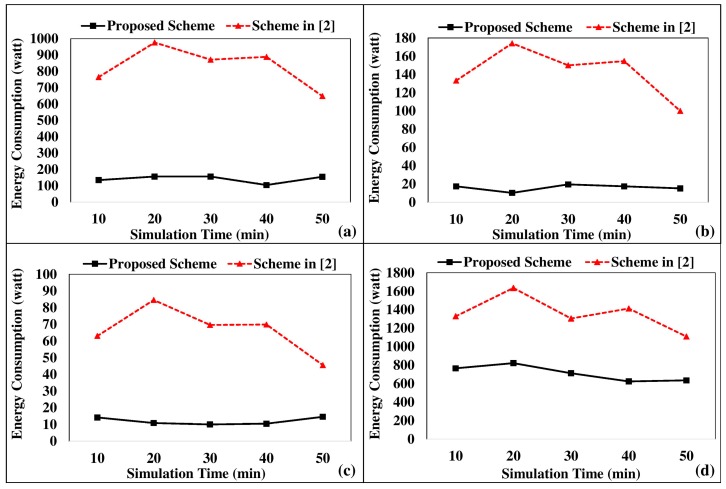
Energy consumption comparison with the scheme proposed in [2]. (**a**) Air conditioner results; (**b**) television results; (**c**) fan results; (**d**) iron results.

Thus, it improves the user comfort level during scheduled operations. Moreover, scheduling of appliances adheres to load balancing restrictions to minimize the monetary expense of energy consumption. As illustrated in Figure 6, the proposed scheme is tested with the energy management scheme proposed in [2]. Figure 6a–d represent the results of an air conditioner, a television, a fan, and an iron, respectively. As illustrated, the energy consumption is significantly reduced in the proposed scheme owing to the automated switching off mechanism, which avoids unnecessary energy wastage. In [2], the switching on and off was purely based on the user context. Nevertheless, the proposed scheme associates a random user initiation supported by an automated switching off context. Subsequently, this approach minimizes unnecessary energy consumption of domestic appliances. In addition, the proposed scheme gives the opportunity to users to monitor and manage energy consumption proactively. In an LST-based scheduling mechanism on top of the cumulative load balancing approach, the cumulative load of the smart home is managed and the appliances are scheduled accordingly.

**Figure 7:**
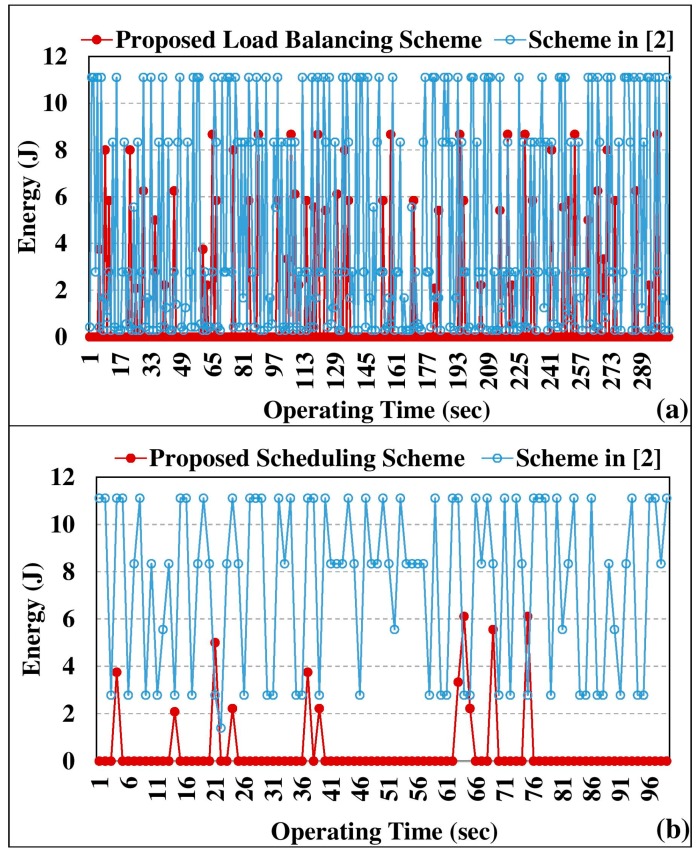
Energy consumption comparison with the scheme in [2]. (**a**) Load balancing energy consumption; (**b**) scheduling energy consumption.

We evaluated the total energy consumption of a household after applying the proposed scheme and the energy management scheme in [2]. The obtained energy consumption results are depicted in Figure 7. Figure 7a reveals that the integration of the switching off scheme has significantly reduced the energy consumption of the respective appliances. The load balancing approach has utilized the aforementioned four constraints to optimize the energy efficiency of a smart household. Since the scheme proposed in [2] manages its services solely on the user context, the energy consumption of the appliances is higher than the proposed approach, which embeds LST scheduling, load balancing constraints, and automated control system. Figure 7b illustrates the energy consumption of three appliances—fan, television, and air conditioner—in the proposed scheduling mechanism and in the energy management scheme in [2]. As depicted in Figure 7b, the proposed LST-based scheduling has reduced the total energy consumption of the smart home compared to the other simulated approach. In addition to the energy efficiency, the proposed LST scheme is modified to operate in multiprocessor scheduling environments. Such that, unlike other existing scheduling algorithms, the proposed work is easily extendable to a smart community that consists of many smart homes.

**Figure 8:**
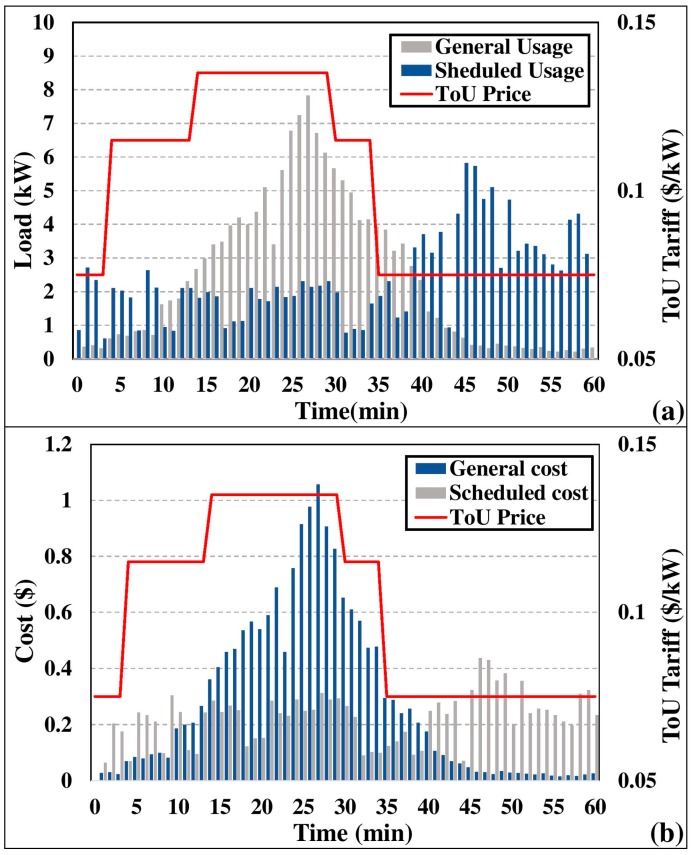
Consumption load profile and cost profile (**a**) consumption load profile of general appliance operation and scheduled appliance operation; (**b**) consumption cost profile of general appliance operation and scheduled appliance operation.

We have evaluated the energy consumption profile and cost of energy for the total household energy consumption. Appliance operation was simulated for 60 min and the simulation replicated off-peak, mid-peak, and peak durations accordingly. Figure 8 illustrates the consumption load profile and consumption cost profile of the household. As clearly presented in Figure 8a, the proposed load balancing embedded LST scheduling algorithm has significantly reduced the peak load demand of the smart home. A peak load of 77.89 kW of general appliance operation was reduced to 28.99 kW, which claims to be a 62.781% peak load reduction. In fact, the peak load reduction influenced the cost of energy consumption during peak hours. As illustrated in Figure 8b, we analyzed the cost profile of energy consumption. Cost optimization of the load balancing component has effectively transferred peak load to off-peak hours with the aid of LST scheduling. The total monetary benefit gained from the proposed work is 20.423% and peak hour monetary benefit is 62.781%, which is similar to peak load reduction.

## 6. Conclusions

Energy management is one of the major concerns in modern smart home implementations. This paper presented an LST-based appliance scheduling scheme that operates on load balancing. In terms of load balancing, the proposed scheme defined four constraints to manage the cumulative energy consumption of a smart home. The scheduling mechanism runs on the load balancing scheme while adhering to the LST algorithm. The utmost goal of employing LST in appliance scheduling was to ensure user comfort during the scheduling process. The LST algorithm was modified to fit a multiprocessor scheduling environment. Thus, it can easily manage multiple smart home scenarios simultaneously. The proposed scheme was tested for energy consumption with the aid of computer simulation. The simulation results clearly reveal the efficacy of scheduling and load balancing with respect to the energy consumption. Moreover, the results depict the superiority of the proposed scheme compared to an existing home energy management scheme in terms of appliance energy consumption.

## Figures and Tables

**Table 1:** Appliance states and descriptions.

State	Description
OFF	The appliance is turned off
ON	The appliance is ready to be operational
FINISH	The appliance has completed the assigned task
OPERATIONAL	The appliance is currently performing a task
SLEEP	The appliance is in SLEEP mode due to an interruption or urgent task
INACTIVE	The appliance is disabled for operations
